# Editorial: Engineering future crops through genome editing

**DOI:** 10.3389/fpls.2025.1720325

**Published:** 2025-10-29

**Authors:** Aytug Tuncel, Hyun Uk Kim, Min Chul Kim

**Affiliations:** ^1^ Department of Plant Science and Landscape Architecture, University of Maryland, College Park, MD, United States; ^2^ Department of Bioindustry and Bioresource Engineering, Sejong University, Seoul, Republic of Korea; ^3^ Division of Applied Life Science (BK21 Four), Gyeongsang National University, Jinju, Republic of Korea

**Keywords:** plant genome editing, CRISPR-Cas, stress resistance, nutritional quality, plant transformation, regulatory policy

Agriculture is facing increasing challenges driven by population growth, climate change, and sustainability demands. In this context, genome editing has emerged as a transformative approach that enables targeted and efficient improvement of crops, accelerating the transition from traditional breeding to precision agriculture.

CRISPR-Cas systems have revolutionized plant biology by allowing highly precise and versatile modification of plant genomes. These technologies are now applied to a wide spectrum of goals, ranging from enhancing resistance to abiotic and biotic stresses to improving nutritional quality, extending shelf life, and minimizing postharvest losses (Tuncel et al., 2023). The CRISPR toolkit continues to expand with the development of new nucleases such as advanced base and prime editors, as well as AI-mediated engineering of novel Cas variants. In parallel, innovations in delivery methods are beginning to address long-standing bottlenecks in plant transformation, including genotype dependence and low regeneration efficiency, with promising results from *in planta* transformation and virus-mediated delivery (Tuncel et al., 2025).

Amid these developments, this Research Topic brings together fifteen contributions that showcase the breadth of advances in this rapidly evolving field. The Research Topic spans multiple themes, including abiotic and biotic stress resistance, nutritional improvement, tool innovation, transformation and delivery methods, and regulatory perspectives. Together, these articles highlight both the progress achieved and the challenges that remain in translating genome editing breakthroughs into agricultural practice. In the sections that follow, we synthesize these contributions and discuss how they collectively illuminate the path forward for crop genome engineering.

## Enhancing stress resistance

Drought and pathogens are among the most significant constraints on global crop yields. Enhancing resilience to these challenges is essential for achieving stable productivity under deteriorating environmental conditions. The five contributions in this Research Topic highlight how CRISPR-based strategies can be applied to enhance stress resistance in diverse crops through both targeted experimental studies and broader reviews of emerging targets and approaches.

Cap-binding proteins (CBPs), which are central to abscisic acid signaling and RNA processing, have been associated with drought resistance in *Arabidopsis* (Hugouvieux et al., 2001) and barley (Daszkowska-Golec et al., 2017). In potato, RNAi-mediated suppression of *StCBP80* improved drought performance (Pieczynski et al., 2013). Building on this knowledge, Decima-Oneto et al. used CRISPR-Cas9 to generate *CBP80*-edited potato lines with enhanced drought resistance. This work demonstrates the potential of genome editing in developing drought-resilient potato varieties, with broader implications for other crops.

Among biotic stresses, downy mildew is a major disease that severely reduces grapevine yield. Giacomelli et al. targeted the *Downy Mildew Resistance 6 (DMR6)* susceptibility genes, which are known to play key roles in pathogen interactions (Thomazella et al., 2021). By simultaneously disrupting *DMR6–1* and DMR6-2, the authors were able to produce grapevine plants with reduced susceptibility to *Plasmopara viticola*, the causal agent of downy mildew. This study shows how genome editing can be applied to perennial fruit crops and highlights the potential for disease resistance by modifying host susceptibility genes.

Complementing these experimental studies, several reviews in this Research Topic provide broader perspectives on CRISPR-based stress resistance. Ton et al. offer a comprehensive survey of CRISPR-Cas applications in *Brassica* crops, covering abiotic stresses like drought, salinity, and temperature extremes, as well as biotic stresses from diverse pathogens. This review highlights promising gene targets and describes a genome editing workflow for developing resilient cultivars. Park et al. further expand this discussion by examining CRISPR-based mutant library screening as a powerful approach to identify novel immune-related genes with a focus on rice and cotton. Chandrasekaran et al. add another perspective by proposing subtilases as genome editing targets to improve yield and quality, citing their roles in immunity, fruit development, and abscission. They present a phylogenetic analysis of pepper subtilases to highlight potential candidates.

## Improving nutritional quality

Beyond yield and stress resilience, consumer-oriented traits such as flavor, allergenicity, and food waste reduction are becoming increasingly important in crop improvement. This Research Topic includes two studies that exemplify these goals, addressing enzymatic browning in wheat and seed protein allergens in soybean.

Polyphenol oxidases (PPOs) drive enzymatic browning, which lowers the quality and marketability of plant products and contributes to food waste. In wheat, PPO activity resulting from grain milling causes progressive browning and discoloration of flour, dough, and other end-products (Taranto et al., 2017). To address this issue, Wold-McGimsey et al. employed a sgRNA targeting a conserved region across seven copies of *PPO1* and *PPO2* in different wheat cultivars. The edited plants exhibited substantially reduced PPO activity, leading to dough with significantly less browning. This study illustrates how genome editing can improve food quality with direct benefits for consumers and food industry.

Soybean allergenicity can be a concern for consumer health. Among soybean seed proteins, GmP34 is considered a major allergen (Helm et al., 2000). Earlier genome editing efforts mainly focused on disrupting *GmP34* (Sugano et al., 2020). Baek et al. expanded this strategy by also targeting the homologous genes *GmP34h1* and *GmP34h2*, which share conserved allergenic peptide motifs with *Gmp34*. Using multiplex CRISPR-Cas9, they generated single, double, and triple mutants with reduced amounts of the allergenic proteins in the seeds. These edited lines provide the groundwork for future allergenicity testing and development of hypoallergenic cultivars.

## Developing novel tools

The effectiveness of plant genome editing relies on advancing the methods and resources that enable precise and efficient editing. Four articles in this Research Topic showcase how innovations in multiplexing, nuclease evaluation, mutational diversity, and computational platforms are expanding the CRISPR toolbox for plant genome editing.

Milner et al. addressed the challenges of multiplexing, a key strategy for targeting multiple genes particularly in polyploid crops. They compared two widely used systems for multiplexing, tRNA processing and ribozyme-based guide delivery, by targeting the same genes in rice, wheat, and barley with identical sgRNAs. Both systems performed similarly in rice, but the tRNA system was more efficient in wheat and barley, providing valuable guidance for multiplexing strategies in cereals.

Building on the need for reliable ways to measure nuclease and sgRNA activity, Cao et al. developed a rapid and accessible hairy root-based assay in soybean. The system uses a ruby reporter for visual identification of transformation-positive roots and was first validated by combining it with Cas9 editing. The authors then applied the assay to engineer and optimize the ISAam1 TnpB nuclease, demonstrating its potential as a compact Cas alternative. Unlike protoplast-based assays, this hairy root platform is simple, doesn’t require sterile conditions, and enables rapid *in planta* evaluation of nuclease and sgRNA efficiency.

In some species, mutational frequency is limited by low transformation and regeneration efficiencies. Ito et al. explored an alternative strategy to generate mutational diversity in tomato by crossing wild type plant with a T0 line carrying biallelic mutations in the *RIPENING INHIBITOR (RIN)* gene. The F1 progeny displayed novel edits absent in the parent, indicating that CRISPR-Cas9 activity can persist beyond transformation and generate additional variation through crossing. This strategy offers a practical option for species or cultivars where primary transformation yields few or no desirable edits.

Complementing these experimental advances, Saraswat et al. review the computational tools in genome editing. They summarize databases and tools used for classification and prediction of CRISPR systems, as well as platforms for gRNA design and off-target analysis. Such resources are essential for expanding the CRISPR toolbox and improving the accuracy, efficiency, and predictability of editing outcomes.

## Delivery methods

Efficient delivery of genome-editing reagents is highly critical in plant biotechnology, particularly for species that are difficult to transform and regenerate (Chen et al., 2022). This Research Topic features three complementary strategies, including viral delivery of compact nucleases, transgene-free RNP editing, and *in planta* transformation methods that help overcome these challenges.

One major limitation of virus-induced genome editing (VIGE) is the restricted cargo capacity of viral vectors, which hampers delivery of large nucleases such as SpCas9. Workarounds include infecting Cas9-expressing plants with mobile gRNAs (Ellison et al., 2020) and using compact nucleases (Weiss et al., 2025). In this context, earlier work with potato virus X (PVX) showed that SpCas9 could induce mutagenesis in inoculated *Nicotiana benthamiana* leaves but failed to achieve systemic editing (Ariga et al., 2020). Ishibashi et al. addressed this by deploying an engineered AsCas12f (about one-third the size of SpCas9) via a PVX vector. This enabled systemic, efficient mutagenesis across infected tissues, demonstrating that compact nucleases can circumvent size limitations and expand the reach of VIGE.

Transgene-free genome editing can ease regulatory hurdles and improve public acceptance. Protoplast transformation with subsequent plant regeneration provides a powerful route, especially for perennial fruit trees with long generation cycles. Citrus is a timely example, as canker disease severely reduces yields. In earlier work, (Su et al., 2023) generated canker-resistant citrus using Cas12a ribonucleoproteins (RNPs) with a single crRNA. In their contribution here, Su et al. extended this strategy by employing three crRNAs targeting *CsLOB1*, the canker susceptibility gene. While the earlier study primarily produced small indels, the multiplex RNP approach yielded long deletions and inversions, demonstrating the feasibility of RNP-based multiplex editing for more complex edits while remaining transgene-free.

Correia et al. review *in planta* transformation methods as alternatives to tissue culture for perennial grasses. Perennial grasses can be highly beneficial for sustainable agriculture because of their potential to reduce soil erosion and improve carbon sequestration, and they require less inputs than annuals. However, their transformation is hindered by genotype recalcitrance and low regeneration efficiency, leaving progress behind other crops. The review explores approaches such as meristem-targeted and virus-mediated transformation, and discusses their potential for genome editing and domestication of these crops.

## Regulatory policies

Although genome editing technologies and applications are advancing rapidly, regulatory and policy frameworks continue to determine how quickly these innovations reach farmers and consumers. Ricroch et al. provide a global overview of field trials, which are essential for assessing the agronomic potential of new traits under real-world conditions, with genetically engineered and genome-edited crops. Their survey shows that research activity is expanding across multiple crop species and trait categories, reflecting strong scientific momentum. Yet persistent obstacles remain, including regulatory delays and, in some regions, restrictive frameworks that slow or prevent field testing. This study highlights the need for harmonized, science-based regulations to ensure that advances in trait engineering move beyond the lab to support sustainable agriculture and global food security.

## Conclusion and future perspectives

The Research Topic *Engineering Future Crops Through Genome Editing* highlights the rapid progress of plant genome editing across traits, methodologies, transformation approaches, and regulatory perspectives. Together, these contributions showcase how CRISPR is being applied to enhance stress resilience, improve consumer-oriented traits, and expand the editing toolbox for diverse crops.

Despite this momentum, barriers such as transformation and editing efficiency, genotype dependence, and regulatory hurdles remain. Moving forward, continued tool development, integration into breeding pipelines, and progressive policies will be essential to realize the full potential of genome editing for food security, consumer health, and sustainable agriculture.
